# BRADSHAW: a system for automated molecular design

**DOI:** 10.1007/s10822-019-00234-8

**Published:** 2019-10-21

**Authors:** Darren V. S. Green, Stephen Pickett, Chris Luscombe, Stefan Senger, David Marcus, Jamel Meslamani, David Brett, Adam Powell, Jonathan Masson

**Affiliations:** 1 Department of Molecular Design, Data and Computational Sciences, GlaxoSmithKline, Gunnels Wood Road, Stevenage, Hertfordshire, SG1 2NY UK; 2 Department of Molecular Design, Data and Computational Sciences, GlaxoSmithKline, 1250 South Collegeville Road, Collegeville, PA 19426 USA; 3 Tessella Ltd, Walkern Road, Stevenage, Hertfordshire, SG1 3QP UK

**Keywords:** Automated design, Cheminformatics, Experimental design, Active learning

## Abstract

This paper introduces BRADSHAW (**B**iological **R**esponse **A**nalysis and **D**esign **S**ystem using an **H**eterogenous, **A**utomated **W**orkflow), a system for automated molecular design which integrates methods for chemical structure generation, experimental design, active learning and cheminformatics tools. The simple user interface is designed to facilitate access to large scale automated design whilst minimising software development required to introduce new algorithms, a critical requirement in what is a very fast moving field. The system embodies a philosophy of automation, best practice, experimental design and the use of both traditional cheminformatics and modern machine learning algorithms.

**Electronic supplementary material:**

The online version of this article (10.1007/s10822-019-00234-8) contains supplementary material, which is available to authorized users.

## Introduction

The search for efficient and effective drug design strategies has been a constant feature of the scientific literature since the concept of rational discovery was introduced by Elion and Hitchings [1–3]. The field of quantitative structure–activity relationship (QSAR) analysis [4–6] developed alongside the rational approach, with the goal of being able to use chemical structures and biological response to develop hypotheses, predictions and design experiments which would provide an efficient path to optimise chemical series into promising drug candidates. Pertinent to this paper, the QSAR community even provided the first example of an automated molecular design system [7], using simplex optimisation and Hansch parameters. Limitations to the—generally-linear statistical techniques employed, use of parameters that were often derived from experiment and insufficient computational power to cope with the combinatorial nature of the chemical space to be interrogated were enough to inhibit the utility of classical QSAR approaches and researchers looked for different approaches to solve their drug design problems.

Protein structure based design techniques have featured heavily in the computer aided drug discovery toolset for more than 30 years, and have had some notable successes in delivering marketed drugs [8, 9]. However, utilising the knowledge of protein–ligand binding interactions to drive the creation of novel, bioactive chemical structures, so-called de novo design [10, 11], has not been straight forward even after 25 years of intense effort. Although 3D methods for design may have dominated, ligand-based QSAR approaches mirrored some of the capabilities [12, 13], for example de novo design from QSAR models (also sometimes known as “inverse QSAR”), and even the first AI-based drug design support system [14]!

In order to establish an efficient and effective computational drug design system, there are a number of fundamental elements that must be constructed:

1. *Molecule ideation*. The generation of chemical structures which are relevant to the problem at hand (i.e. lead optimisation), are synthetically tractable and do not contain structural liabilities.
2. *Prediction*. The generation and application of computational models which cover the entire Target Compound Profile (TCP: primary target, selectivity, ADMET, physicochemical attributes). It is likely that the suite of models will include both statistical/QSAR models and physics-based models [15].
3. *Selection*. From the list of ideas generated and evaluated with the model suite, which ones should be made next in order to meet the TCP, or more likely, to generate knowledge that will best enable the project to design a molecule which does meet the TCP.
4. An infrastructure that will enable these elements to be coupled together in a robust, efficient manner and can handle the likely large numbers of chemical structures produced.

Fortunately, the science of cheminformatics [if this is broadly defined to encompass chemical databases, chemical structure generation (Q)SAR modelling, experimental design and the application of machine learning methods to all three of those areas] has advanced to the point where credible solutions exist for all of the fundamentals and it may be possible to join them together. That this was indeed possible was illustrated by the first example of a fully automated design project, which started with an approved acetylcholinesterase inhibitor drug and created brain-penetrable ligands with either specific polypharmacology or selectivity profiles for different G-protein-coupled receptors [16]. Several groups have published examples of automated design [17, 18], often coupled with automated synthesis/test systems [19] which are well suited to pilot this new design paradigm [20].

The emergence of Deep Learning methods has quickly added a variety of techniques for QSAR modelling [21–25], molecule ideation [26–31] and synthetic tractability [32, 33]. The rapid rise of publication volume in this field indicates that more is to be expected and recent suggestions for standard benchmarks [34, 35] are welcome.

It is not only Deep Learning methods that can effectively utilise large SAR datasets. Knowledge based cheminformatics methods are able to mine and reapply chemical transformations in the form of Matched Molecular Pairs, MMPs [36–42], and automatically discover SAR patterns and suggest new substituents to optimise a biological response [43–45].

From the perspective of an industrial group, the rapid development of new algorithms and capabilities may provide a step change in our ability to generate and evaluate large numbers of novel chemical structures. The challenge is to build a system which can integrate multiple approaches from disparate disciplines, make it robust enough to be used by multiple people across a portfolio of projects, make it simple to add or remove algorithms and make the tools accessible without repeated training as new code is added. Because the new algorithms are capable of generating very large numbers of ideas that will overwhelm a “selection by visual inspection” approach, a different way of working must be adopted. Here there are opportunities beyond just using new algorithms: to build in best practices (e.g. safety alerts [46–48], physicochemical properties [49–51], multi parameter optimisation), to automate the expert so they might spend more time on harder problems or work on more projects simultaneously and to greatly reduce the time and resources expended on end-user tools and training. This last point, to reduce the amount of time medicinal chemists spend using tailored computational software, might be considered controversial. However, a recent study from the banking sector [52] found that return on investment to corporations from end-user software development is often marginal, whilst there was significant value derived from investments in automation, and in a modern world where a virtual assistant can order your shopping via speech recognition, it is surely reasonable to be able to ask “Alexa, which are my best R groups?”.

In this paper we describe a solution to this challenge: BRADSHAW (**B**iological **R**esponse **A**nalysis and **D**esign **S**ystem using an **H**eterogenous, **A**utomated **W**orkflow), a system for automated molecular design which integrates methods for chemical structure generation, experimental design, active learning and cheminformatics tools. The project was inspired by a paper [53] which describes a system through which “A computer language, ALEMBIC, is used to collate the ideas of the scientists. The resulting list of potential molecules is then parametrised using whole molecule descriptors. Based on these descriptors, appropriate statistical techniques are used to generate sets of molecules retaining the maximum amount of the information inherent in all possible combinations of the scientists ideas”. If an algorithm can be considered as an additional scientist, BRADSHAW can be considered as a direct descendant of that heritage Glaxo system.

## System Architecture

The BRADSHAW system is an integration between a number of external components. Its primary purpose is to orchestrate the running of data pipelines over compound sets (“Tasks”) and chaining the inputs/outputs of these Tasks to form designs.

The BRADSHAW system uses Docker containers to integrate various data management Tasks and the UI. The User Interface is implemented using Angular CLI (version 1.1.1) [54].

The Tasks are described by an interface to a web service with a set of parameters, with the expected columns in input and output files also defined. This means that an administrator can easily create new Tasks without redeployment of the system. The administrator defines the parameters for a Task using a form in the user interface. This generates a form from which users can use these Tasks to build their design workflows for lead optimization with their chosen parameter values. An API interface is then imposed on web services when implementing the Tasks in order to integrate with BRADSHAW. The choice of technology for the web services is not imposed by the system; however typically these are built in Pipeline Pilot [55]. However, the BRADSHAW system itself knows nothing about Pipeline Pilot and any web-service could be used. Request parameters are passed in a single JSON object so that other web-services can be written if desired. We have developed utility components to read and validate the request JSON and to make it straightforward for developers to integrate with the call-back functions. The requirements for any protocol are minimal. Essentially the protocol must interpret the request JSON and send a call-back message to a defined url once complete. Status updates can also be sent using the call-back. This provides a way to give users feedback in the UI for long running Tasks.

The input of compound sets is from integration with either with LiveDesign [56] or via a file upload. Users can view the content of any files whether inputs or intermediate results in a design to review the work as they go along. This includes the handling of large compound sets via file streaming.

A user constructs a design by sequential addition of Tasks to a workflow and therefore can deploy as few or as many computational steps as they require. Some Tasks have restricted connectivity, which is governed through the required inputs/outputs of the files of each strategy. This enables us to impose best practice where we feel it is justified (for example, the Molecule Generator must always be followed by a Filter step to remove structures which do not meet agreed standards). Design workflows then, by definition, are validated. For simplicity, the designs are made up of a linear chain of Tasks. However, when a user wants to reuse the output of one part of a design they can use that as the input to multiple new designs, this permits branching flows.

**Fig. 1:**
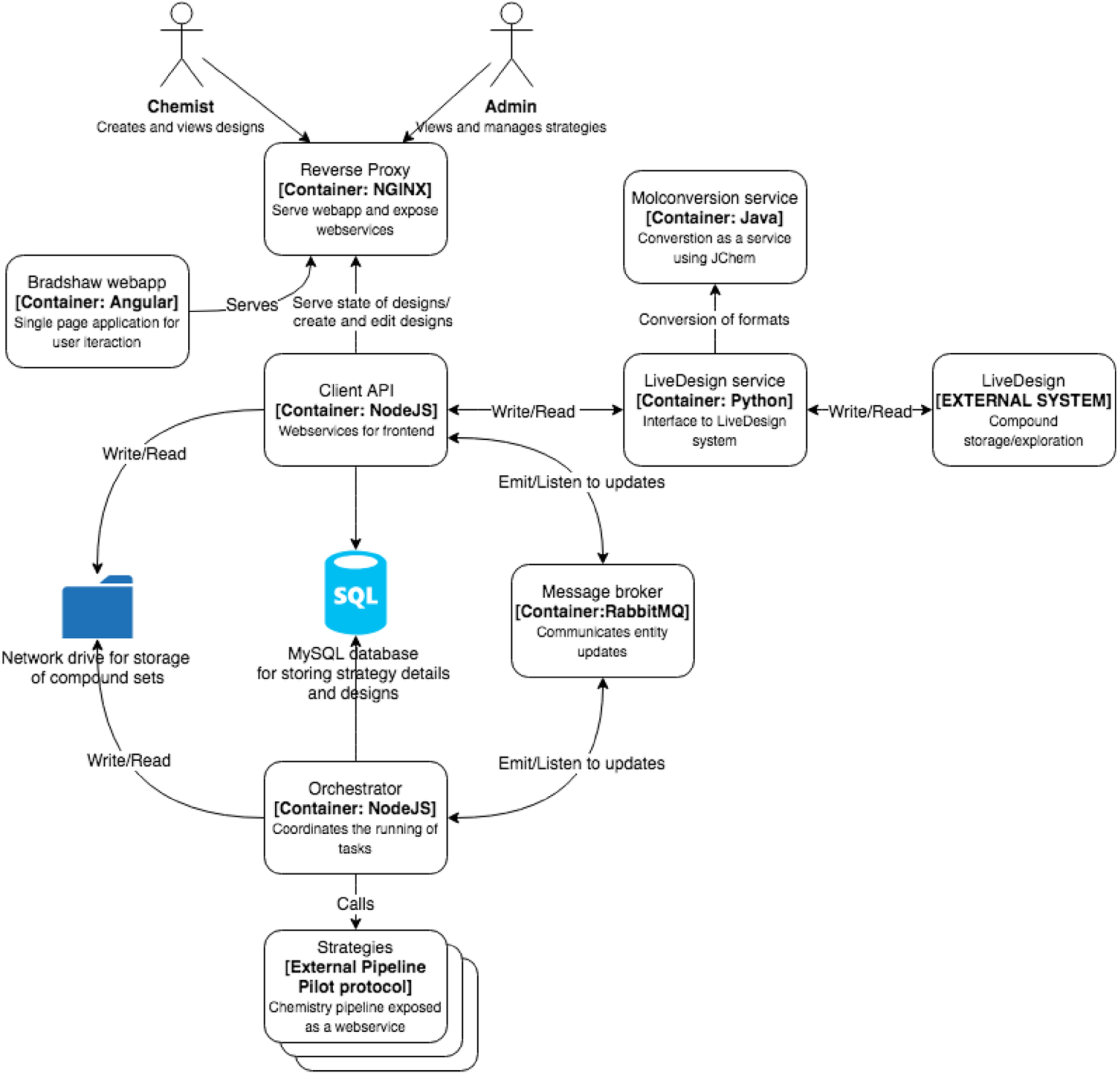
Overview of the BRADSHAW system

Once a user runs a design there is immediate feedback to the user via web sockets allowing them to view the progress and logs from strategies and see the intermediate results as they are produced. There is also constant feedback on the health of running strategies via a beating heart in the user interface. The active feedback is enabled via a message queue in the system to push the status updates back to the user (Fig. 1).

A number of Tasks have been implemented and these are described in the next section.

## Tasks

**Fig. 2:**
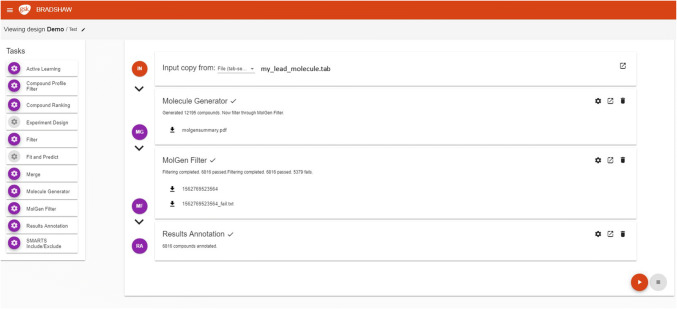
The BRADSHAW User Interface. Available Tasks are on the left, and are coloured if they can be added to the current workflow

In BRADSHAW a Task is the term used to identify a particular scientific process. A Task may be complex and long running or relatively simple and fast. However, it performs one particular function, for example generating molecules from a lead compound, filtering generated molecules on properties, filtering molecules on substructure, selecting a subset of compounds using experimental design, preparing a file for analysis. The options for running a Task are configurable through the BRADSHAW UI (Fig. 2). However, one of the main design criteria is that the Task should encapsulate “best practice” by default. Extensive user configuration is not a primary design goal, for example, the Compound Molecule Filter contains defined settings for Oral and Inhaled drug-like properties. The user can provide their own XML configuration file to override these defaults but that should be manipulated outside of BRADSHAW.

All Tasks are implemented as Pipeline Pilot protocols and many are run standalone in other applications with the BRADSHAW protocol wrapping existing protocols or components.

The system is designed to make the deployment and integration of new scientific Tasks as easy as possible. Because the area of molecule design and machine learning algorithms is in a period of rapid change, we fully expect our current range of Tasks to have a high rate of turnover, or at least be modified to reflect progress in the scientific literature and experience of application in real projects. BRADSHAW will enable us to make cutting edge science accessible and integrated with other necessary workflows, with minimal software development or end user re-training. We have both test and production systems, so new Tasks can be thoroughly evaluated before being pushed to the production environment.

### Molecule generation

The main purpose of this Task is to generate compounds from one or several lead molecules. At present this Task is focussed on lead optimisation, with relatively modest changes made to the lead molecules (a future goal is to add a “lead-hop” molecule generator, which may include additional generative methods). The methods implemented at the moment are a combination of established cheminformatics methods and more recent deep generative models. However, this Task is the poster child for a fast moving area of research, and we expect to add or replace methods on a regular basis.

The user does not control which methods are run. However, there is an option to limit the output by similarity to a user-defined pool of compounds either by excluding or including generated molecules that are similar. Different criteria are defined for either case as explained below.

Molecule generation methods available currently are:

(A) *MMP transforms*. We have an extensive collection of molecular transforms captured in the GSK BioDig system constructed using the algorithm of Hussain and Rea [37]. The input molecule is fragmented according to the fragmentation rules and BioDig is searched for relevant transforms. These transforms are then applied to the input molecule in a sequential fashion. For example a phenyl ring may be changed to a heterocycle or substituted with a methyl group.
(B) *BRICS*. BRICS is a methodology developed to fragment molecules at defined chemically labile bonds [57]. This extends the well known RECAP approach [58, 59] with some additional disconnections and has been implemented in RDkit [59]. As implemented in RDkit BRICS works by fragmenting molecules and then recombining the fragments in an iterative fashion. As the fragments are recombined randomly and only to a predefined depth the output molecules can bear little resemblance to the input molecules. For our use case we prefer to modify the input molecules in a systematic fashion. Thus the RDkit implementation has been extended in Python to provide additional methods that provide greater control over the generation phase, removing the random combinations in BRICS and imposing a sequence of recombination which ensures that application of the procedure will generate molecules with similar topology to the input (and has the reassuring feature that a fragmented input molecule will always regenerate the input molecule!). In a separate process the BRICS fragmentation has been applied to the GSK collection. The fragments are collected into a pool according to the atom labels that indicate possible recombinations with frequency and molecular properties (heavy atoms, molecular weight). The modified algorithm is used to generate structures by randomly or exhaustively replacing a particular fragment in the input molecule with fragments from the pool that share the same labels, so generating new molecules for further evaluation. The BRICS bond definitions are very specific for certain atom types, ring types and substitution patterns so options are given to allow some flexibility when recombining. For example, BRICS differentiates bonds to aromatic and aliphatic rings. An option has been added to remove this restriction when combining fragments thus allowing a phenyl to be replaced by cyclohexyl. Fragments can also be inserted (allowing for compound growth) or null fragments used to remove a fragment.
(C) *Similarity searching*. Input molecules are searched against a number of internal and externally sourced datasets of available compounds, GSK collection, eMolecules [60], EnamineREAL [61]. Searches are performed using the MadFast application from ChemAxon [62] which provides results in seconds even for the largest databases. Two different fingerprints are used: ChemAxon path-based fingerprint 2048 bits, path 7, 4 bits per feature (CFP7) and the ChemAxon implementation of ECFP4 [63] with appropriately defined cutoffs [38].
(D) *RG2SMI*. A deep generative model that generates molecules that have the same Reduced Graph as the input molecule [27]. Up to 1000 molecules are generated per input molecule.

As mentioned above there is an option to filter compounds according to similarity to an external pool of compounds or the input molecules. If the desire is to filter to similar compounds then a number of similarity measures are used: Tanimoto similarity using CFP7 and ECFP4 fingerprints and identical Reduced Graph [64]. The use of multiple techniques is to ensure all molecules of potential interest are carried forward. Where the user wishes to exclude similar compounds just the CFP7 is used at a tighter threshold, to ensure that only close analogues are excluded.

### Compound profile filter

Multi parameter design is built in to the system. Compounds are filtered according to a set of property criteria as appropriate to the program objectives. The default options are preconfigured for Oral or Inhaled compounds. The user can provide a custom configuration by supplying their own XML file. A large range of molecular properties, predictive models for ADMET liabilities, permeability, solubility are available through GSK internal web-services, QSAR Workbench models [65] or as native Pipeline Pilot descriptors. The Task also runs GSK specific substructural filters to identify undesirable chemotypes [66]. It is a requirement of the system that the molecule generator output be processed by this Task. The intention is that the system never designs a molecule that is not predicted to be compatible with the program objectives, and does not produce molecules that contain chemical structure liabilities known to the organisation.

### Compound rank

Compounds are ranked according to a user-defined multi parameter scoring algorithm. The properties and applicable ranges are defined via an XML file (an example file is provided in the Supplementary Material). The ideal score for any property is 1 and decreases linearly from the defined min/max according to the gradient. Scores for all properties are summed and compounds are ranked according to the score. Alternatively the compounds are ranked according to the Pareto rank of the scores. Optionally the output can be limited to the top N compounds (with Pareto this will return all compounds from the appropriate Pareto front so more compounds may be returned).

### SMARTS filtering

The program may be interested in particular chemotypes or indeed in avoiding particular substructures. This Task allows user-defined SMARTS to be entered and used to either include or exclude matched compounds. SMARTS filtering is implemented using the ChemAxon java toolkit.

### Merge

A relatively simple and important Task. This allows addition of chemical structures which have been generated outside of BRADSHAW [for example, an enumeration of compounds that can be made using an available intermediate and accessible building blocks, or a LiveReport (the LiveDesign term for a user defined set of molecules and data) which is the result of a medicinal chemistry brainstorm session]. In this way, the machine and human generated ideas can be processed with the same workflow and subjected to exactly the same selection methods. All ideas are still equal and human creativity is added to the machine.

### Active learning

A key component of the system is the ability to incorporate knowledge from previous rounds of screening and to suggest the most appropriate compounds for the next iteration. Active learning [67] provides a framework for such an approach. We have implemented an Active Learning protocol based on the modAL Python library [68]. A set of molecules, most likely derived from the molecule generation and filtering Tasks described above is used as the pool and a second set of molecules with measured activity data is defined for model building. The Task automatically builds and validates a QSAR model and uses it to predict the activity of the molecule pool. The output is a list of suggested molecules for synthesis at the next chemistry iteration, annotated as to whether the molecule was selected to “Explore” or “Exploit” from the QSAR model. The current Task uses Random Forests or the XGBoost variant, where the uncertainty in voting patterns in the trees is used to define if a molecule is in the “Explore” or “Exploit” category. The user has control over how much Exploration is performed in the design step, as at present we do not have sufficient experience to set these automatically. Ideally this balance between Explore and Exploit would be set algorithmically and is the subject of current research.

### Experimental design

At the start of a program or when exploring a new series there may be insufficient data to initiate an Active Learning cycle. We have implemented an experimental design Task that provides an efficient and informative mechanism for selecting compounds for a first iteration of lead expansion.

BRADSHAW utilises Design of Experiments (DOEs) based approaches for exploratory chemical array scenarios where the full (M × N) array cannot be synthesized for practical reasons. By treating each monomer in the array as a categorical factor of the design, a balanced fractional array design can be generated. Once synthesized and measured, the results can be statistically analyzed to assess the additivity of SAR and then determine the contribution of the monomers to potency, selectivity and other properties of interest. This novel approach can be successfully used to understand and exploit the SAR of a late stage lead optimization program.

The approach combines well established experimental design techniques as a first step to achieve a well balanced design scheme. The appropriate scheme is utilized in a second library optimization step that allows the incorporation of whole molecule properties or other considerations into the choice of final products. In the standard workflow the user defines a combinatorial array of molecules for synthesis from available building blocks using design tools outside of BRADSHAW such as LiveDesign. LiveDesign is used to define the scaffold(s) and R-group definitions and the annotated LiveReport used as input to the Task.

To create an experimental design each R Group position must be treated as a categorical factor and each monomer at that position as a categorical level of that factor. The objective of the Design generation is to sample across this categorical feature space in such a manner that a robust and objective assessment of the monomers’ contribution to the response can be made. For the purposes of a Medicinal Chemistry Lead Optimization problem we only need two or three factors but the ability to specify large numbers of levels per factor is required. The experimental design that produced by BRADSHAW is an equally sampled, incomplete balanced block design (colloquially known as a “sparse” array) in which each monomer at a particular position is treated equally, i.e. it is sampled the same number of times as others in that position. The sampling rate will be different at different R-Groups unless the number of levels is identical.

**Fig. 3:**
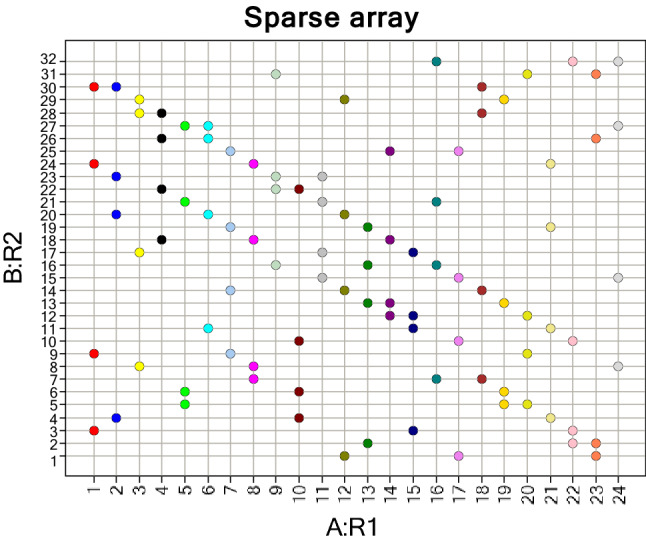
Example of a sparse array design for a two component library

Once the number of levels has been specified at each R Group position the only decision that needs to be made is the size of the fraction to be sampled in order to explore the categorical feature space. For example, if RG1 has 24 monomer levels and RG2 has 32 monomer levels then it is possible to extract a 1/8th fraction in which each RG1 monomer is chosen 4 times and each RG2 monomer is chosen 3 times. The number of compounds sampled is 96. This is shown visually in Fig. 3 where each spot represents a compound to be synthesized from this virtual array.

The Pipeline Pilot protocol uses R to build either an equal sampling design (two R-group positions) or a factorial design for the defined number of compounds. The output from the design is essentially a list of coordinates in the dimension of the virtual library. The designs themselves are agnostic to the actual characteristics of the individual reagents. They only know the dimensionality of the problem and the number of items in each dimension. Thus, the user needs to assign actual physical reagents to each position in the final design. This problem has been addressed in keeping with the automated philosophy behind BRADSHAW. We have developed and implemented a novel library design algorithm that is completely generic in nature in that the algorithm makes no assumptions about the shape of the design, combinatorial or non-combinatorial. The user defines the product map as a set of coordinates within the virtual library based upon the chosen experimental design, specifying which points of the virtual library should be included in the design. The algorithm assigns reagents to these positions in such a way as to optimize user-supplied product-based properties for the resultant library, starting with random assignment followed by a steepest descent optimiser.

### Fit and Predict

The SAR generated from a Sparse Array is ideally suited for a classical Free-Wilson analysis [69]. In keeping with the automated philosophy, the Fit and Predict Task is used to both build the QSAR model and predict the activity of all combinations of R groups in the data set.

### Results annotation

A lesson learned in our pilot projects is that it is most important to annotate the selected compounds in order to facilitate a smooth project team discussion:

- What parts of the structure are being modified? Annotating in this way can integrate the output of a machine-driven process back into the thought process of a medicinal chemist and therefore aid assimilation and acceptance of the ideas.
- What are the predictions and what is the confidence?
- What is the likely synthetic tractability? Note that at this score is used as an annotation, rather than a filter, because none of the published methods perform well enough on our in-house data for them to be considered predictive. The current implementation uses the SCScore [33] algorithm. At some stage in the future we expect that this will change, and at that point the synthetic tractability would be moved further up in the design workflow, either within the molecular generators or as a compound filter.
- More general annotation is based on the GSK SIV framework [70]. Compounds are clustered using several methods: sphere exclusion clustering, framework clustering [64] at different levels. Compound properties are computed such as PFI [51].

## Examples

### Molecule generation of adenosine A2A antagonists

**Scheme 1:**
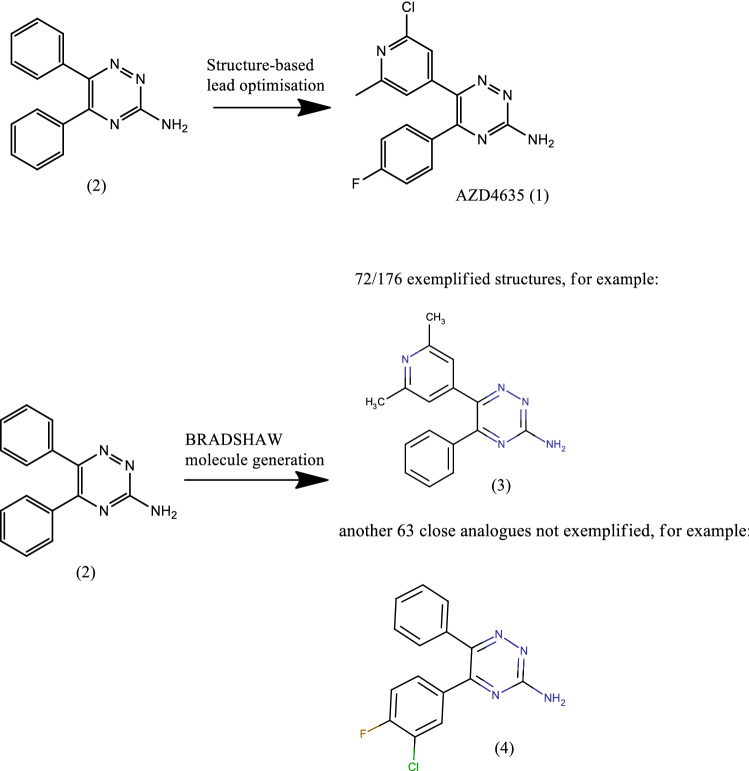
Molecule generation for Adenosine A2A antagonists

The discovery and optimisation of Adenosine A2A antagonists [71, 72] is the first example of successful structure based design using crystal structures of a GPCR. The discovery of the clinical candidate, AZD4635 (compound 1), was based on an initial hit, the commercially available 5,6-diphenyl-1,2,4-triazine-3-amine (compound 2). Optimisation was driven using crystallography, computational methods and state of the art medicinal chemistry thinking. 176 Chemical structures are exemplified in the Heptares Patent [73], as retrieved from SureChembl [74]. BRADSHAW was fed 5,6-diphenyl-1,2,4-triazine-3-amine as an input and using a single pass of molecule generation (only making changes to the one starting structure), filtered according to our Oral Drug protocol and keeping the 1,2,4-triazine-3-amine headgroup, produced 10,675 structures. 72/176 of the exemplified structures were generated and a further 63 close analogues (ECFP4 Tanimoto > 0.8) generated which were not exemplified in the patent (Scheme 1). The BioDig molecular generator produced a minority of the ideas (317 structures), but these were very efficient in mapping to the patented structures (29 exact matches). In a real-world application, this set would be added to by further molecular generation after the first SAR is generated. However, we believe this small example is a good demonstration that BRADSHAW generates the “right” kind of molecules, which are both relevant to the project and synthetically tractable.

### Experimental design of MMP-12 inhibitors

**Scheme 2:**
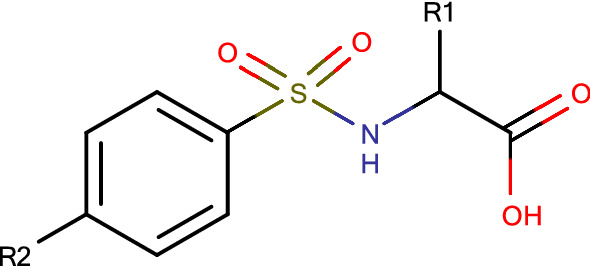
The core template for a 50** × **50 array targeted against MMP12

A recent publication [18] disclosed a full 2500 member combinatorial library with associate biological data. The dataset is particularly relevant as there are a number of compounds that could not be synthesized successfully. The library was based on a core template as shown in Scheme 2.

With such a complete array it is straightforward to identify the most potent compounds and the key monomers which are contributing to improved assay performance. The question that one could ask is whether the key findings from this particular chemical array could be obtained from only a fraction of the compounds?

Using BRADSHAW, an incomplete balanced block design is generated where by each of the 50 monomers in each position was selected twice and only twice in the design. This produced a design in which 100 compounds were sampled from the 2500 array set. The balanced nature of the design meant that each monomer was equally leveraged and would thus in theory allow an unbiased assessment of the monomers potential to add or subtract value in terms of the assay response.

The created design pairs up monomers in R1 and R2 but the assignment of which monomer is allocated to which labelled Level can either be done randomly or by using an algorithmic approach to optimise the final assignment so as to produce a sampled set of compounds which are more optimally selected against a chosen property, e.g. lipophilicity, predicted permeability etc. For the purposes of this exercise we optimised the final selection to meet the design constraints (each monomer chosen twice and only twice) and also to maximise the leadlikeness of the molecules using a desirability function which combines counts of hydrogen bond acceptors, donors and rotatable bonds, clogP, PFI, polar surface area and predictions of hERG and p450 inhibition.

Once the final compound set had been identified their respective potency values were retrieved from the full 2500 array data set. 64 Data records were found with measured potency data from the 100 compounds identified for synthesis. This is in line with the overall attrition rate in the completed array. A selection of the more interesting compounds is shown in Table 1, with the full list available in the Supplementary Material.

**Table 1:**
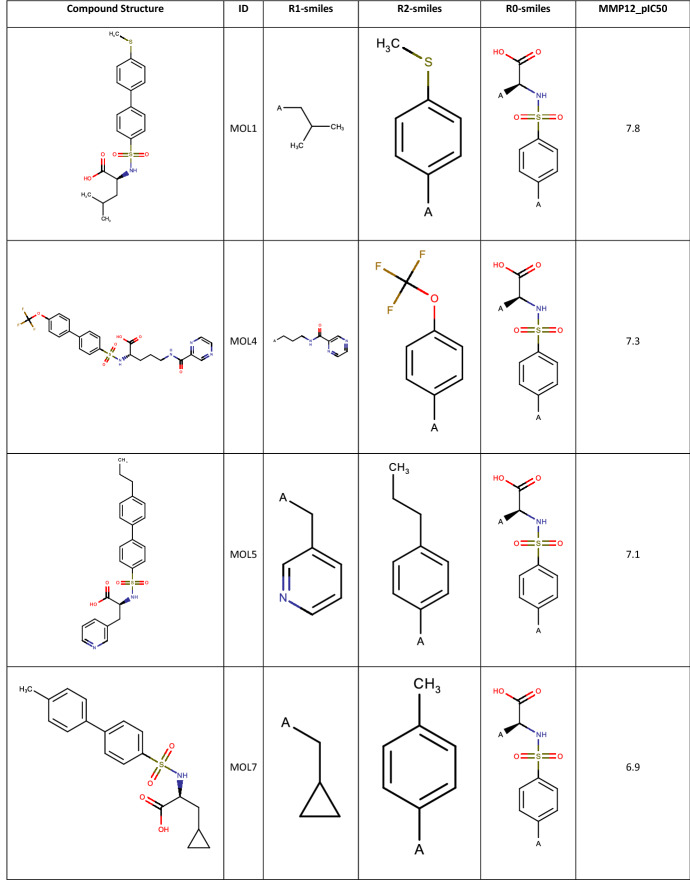

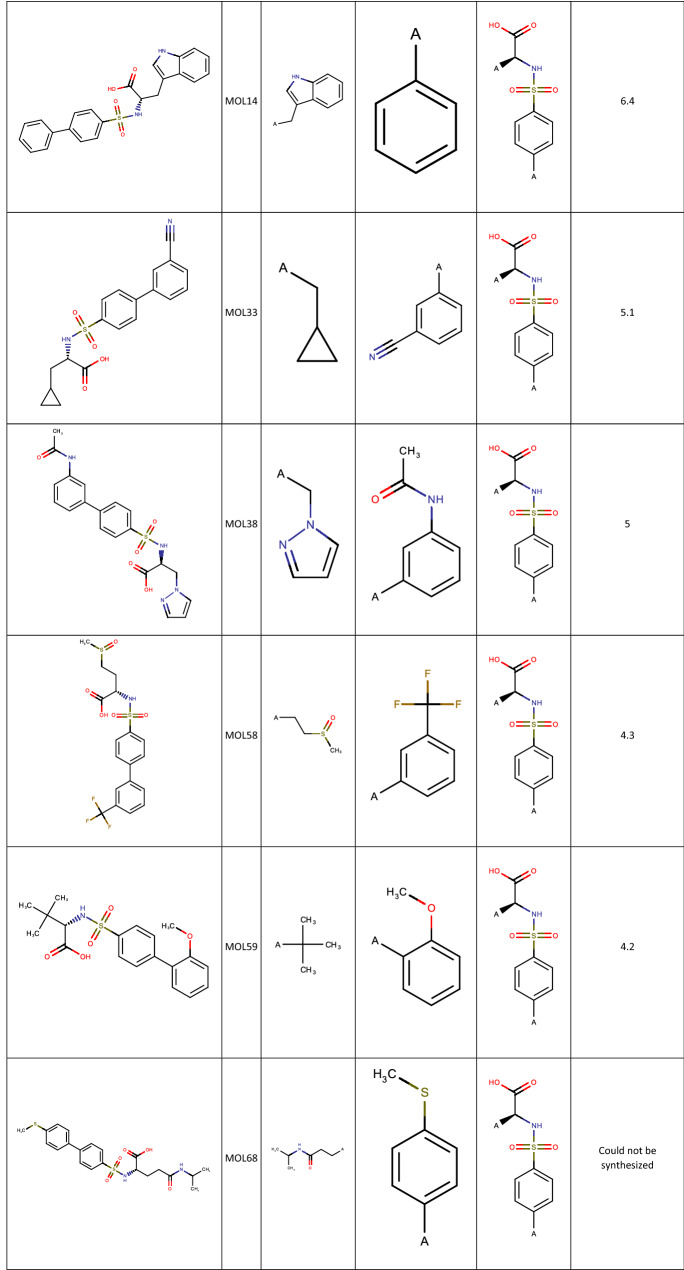
Compounds selected from the MMP12 Sparse Array design along with their biological data

Using the Fit and Predict Task, a Free-Wilson QSAR model was built and the MMP12 activity of the non-selected members of the full array were predicted. The top 10 predictions are shown in Table 2.

**Table 2:**
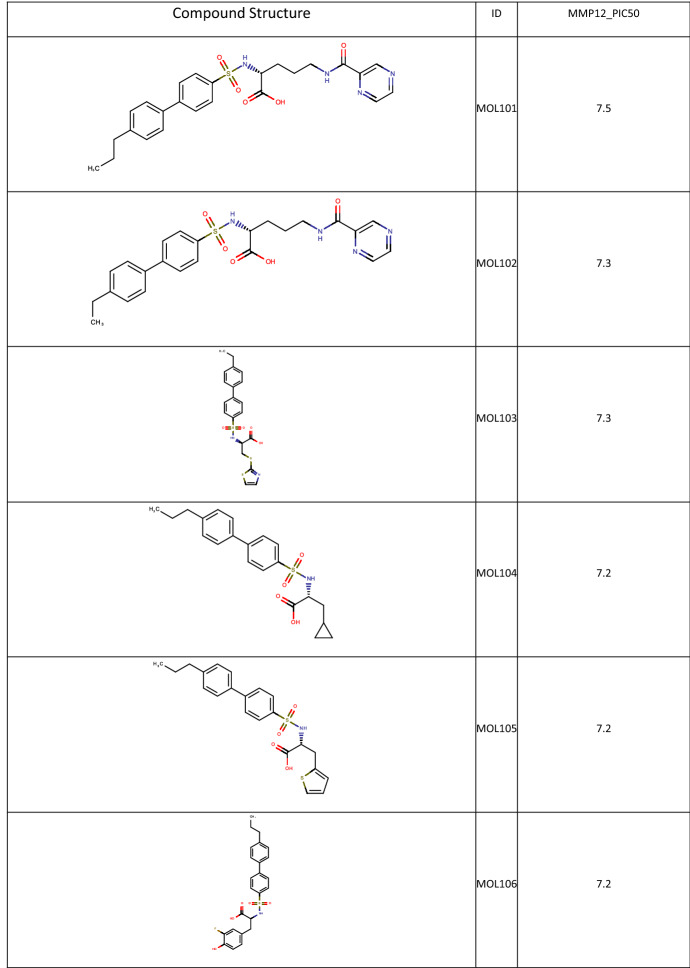

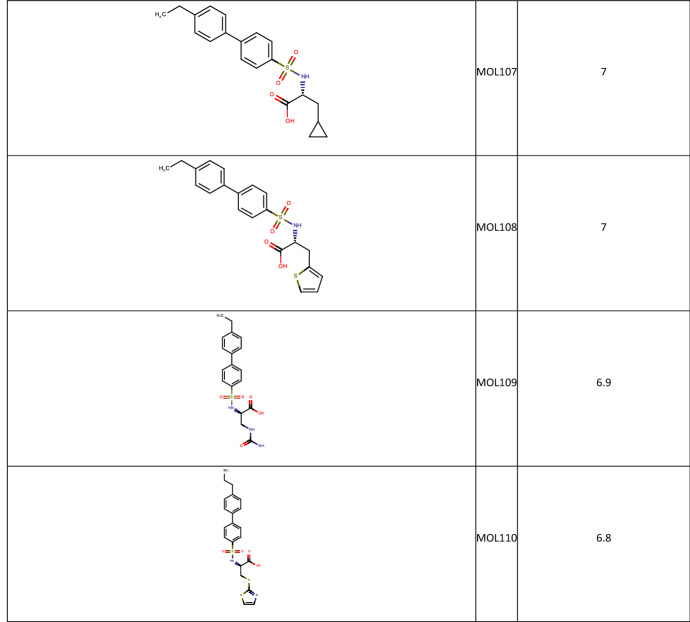
Compounds from the MMP12 set predicted to be the best actives using the Fit & Predict Task, along with their MMP12 activity

**Fig. 4:**
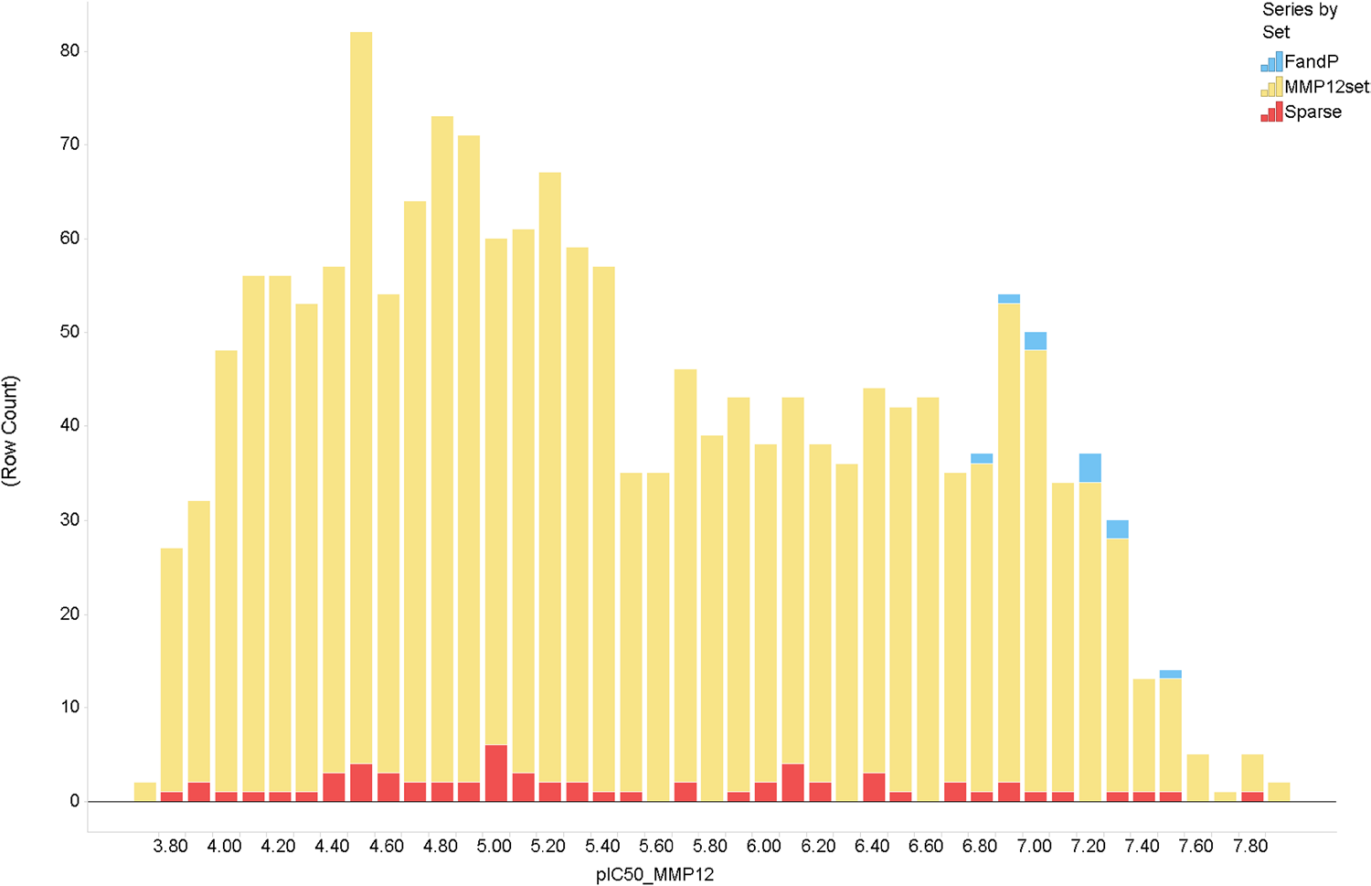
MMP12 activity distribution across the MMP12 data set (yellow), the sparse design (red) and the Fit and Predict selections (blue)

The designed sparse array represents just 2.5% of the full array and the Free-Wilson model is built on just two exemplars per monomer position. From this design, 8/10 of the top predicted molecules (that could be synthesized) have a pIC50 > 7. A comparison of the MMP12 activity for the whole data set, the sparse design and the Fit and Predict selections is shown in Fig. 4.

### Active Learning of MMP12 inhibitors

Active learning is a powerful technique which is suitable to guide iteration experimentation such as Lead Optimisation and is a natural follow on to an initial Sparse Array design. As a demonstrator and comparator to the Fit and Predict example, the output of the MMP12 Sparse Array was used as a seed for a BRADSHAW Molecule Generation and Active Learning workflow. The top 5 most active compounds from the Sparse Array were used as seeds for the Molecule Generator. 53,707 molecules were generated, filtered using the Oral molecule profile and a SMARTS query to only consider molecules with the phenyl-sulphonamide-acid core. Those molecules from the full MMP12 set that were not part of the sparse array were Merged, giving a total of 7385 compounds. These were fed as the selection pool to an Active Learning Task, which used the MMP12 pIC50s from Sparse Array design as the training set. An XGBoost model was built with 100 trees, and the Active Learning was asked to select 50 molecules, with 80% Exploit and 20% Explore (chosen because the QSAR model from a spare design is generally good, favouring Exploit, and our experience with Active Learning is that some Explore is always a good idea). A selection of the resulting molecules are shown in Tables 3 and 4. 26 of the compounds selected are present in the experimental MMP12 data, including one of the most potent compounds at pIC50 of 8. For the generated structures, the best biphenyl substituents (CS- and propyl-) are selected amongst the molecules and the SAR around alkyl amino-acids is sampled in some detail.

**Table 3:**
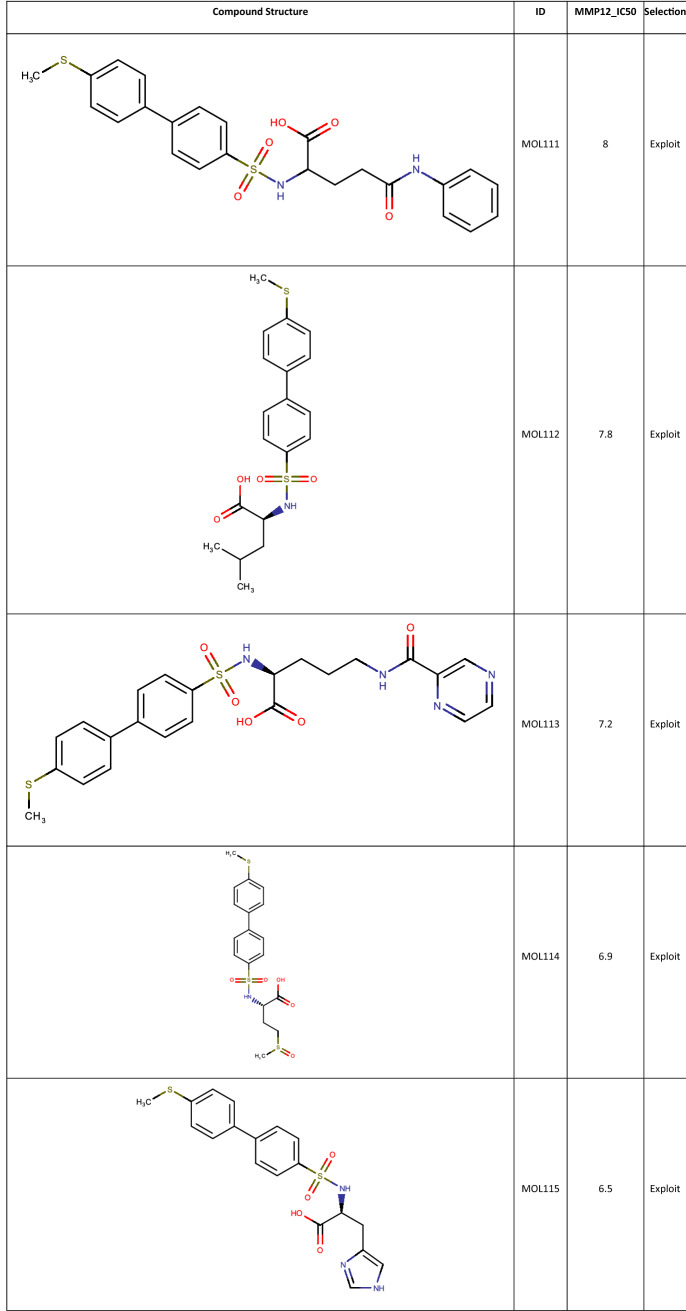

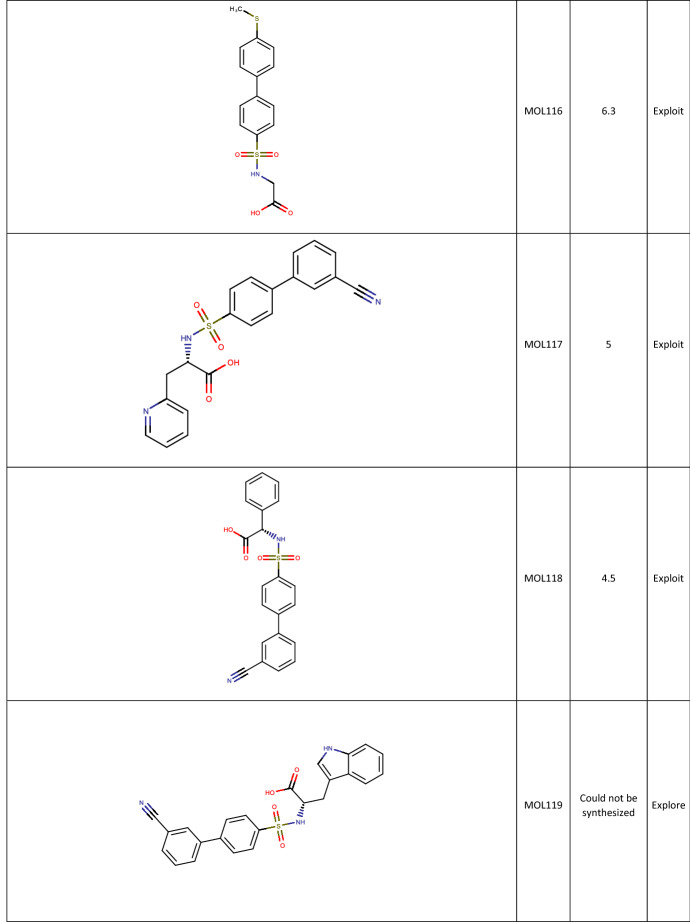
Molecules from the full MMP12 data set selected by the Active Learning Task

**Table 4:**
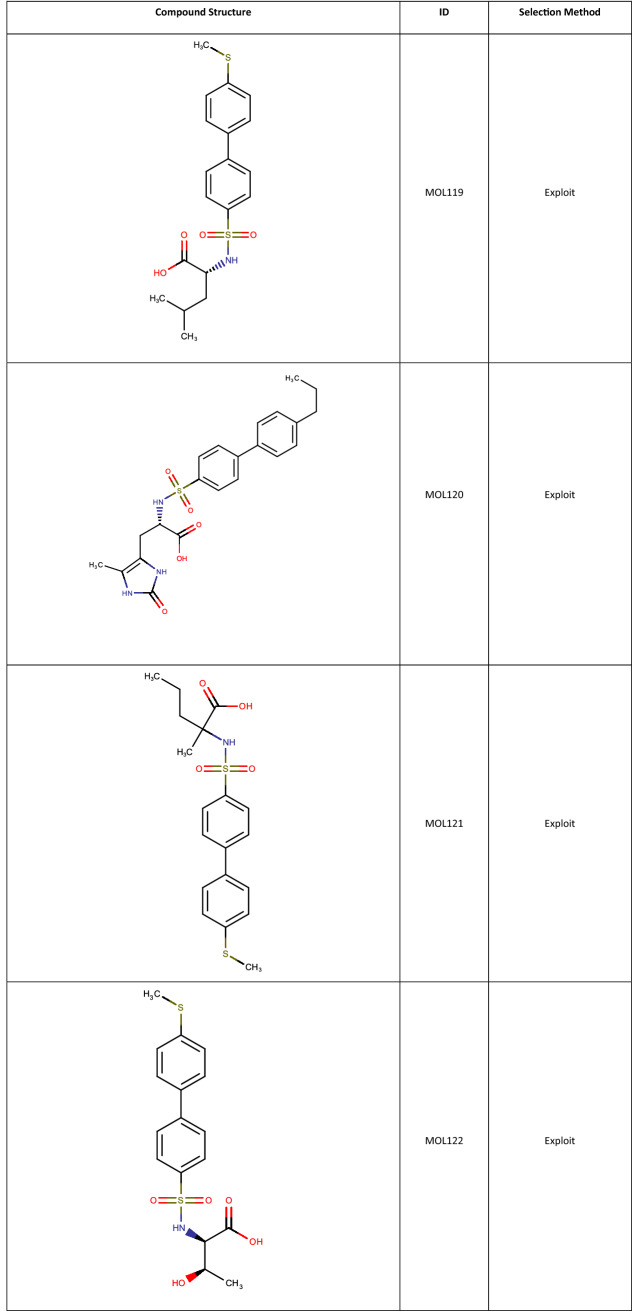

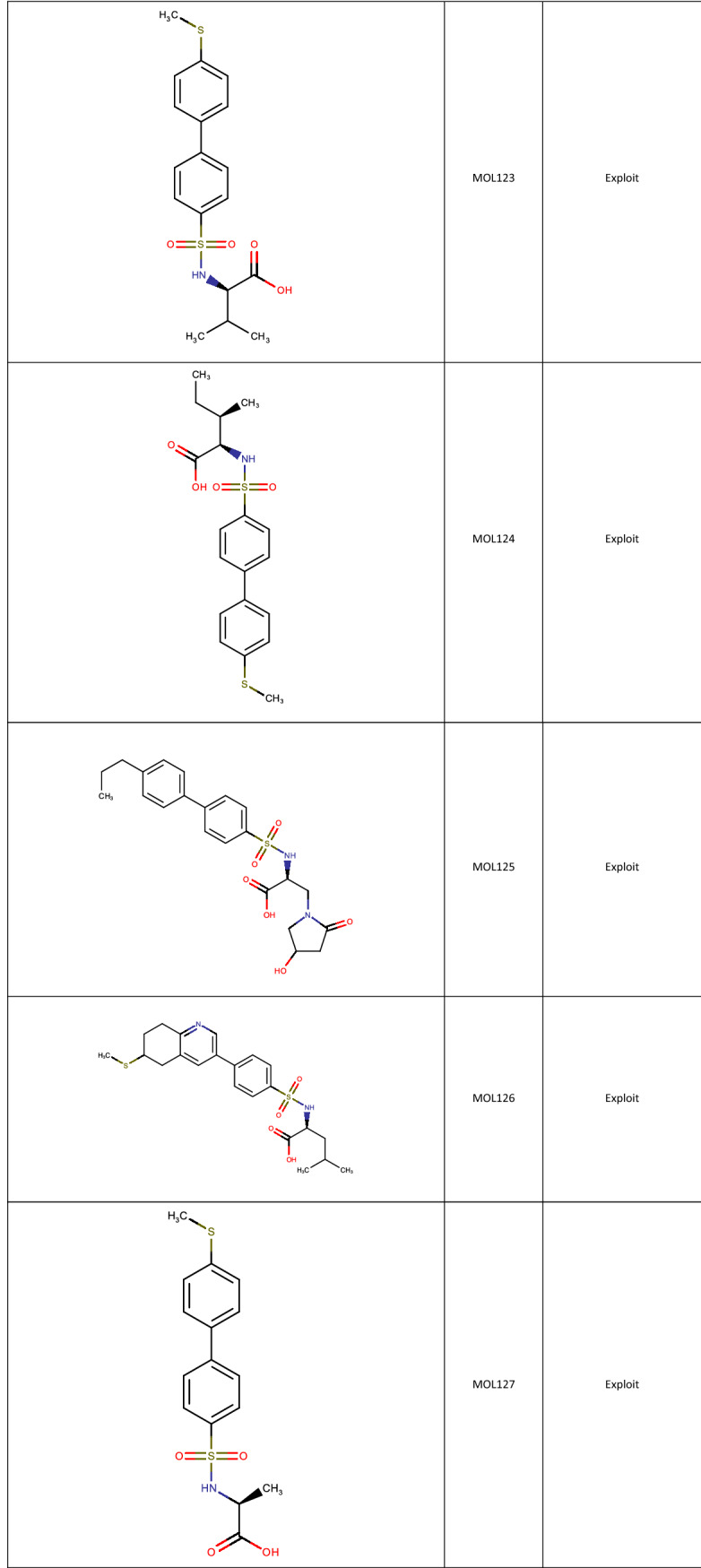
Molecules generated by BRADSHAW and selected by the Active Learning Task

## The future

In the current implementation, BRADSHAW is limited to cheminformatics and machine learning models. There are no 3D or docking methods, partly because it is difficult to produce a standard Task that can cater for the more bespoke models typically required in that domain, and partly because the predictivity and uncertainty quantification is not yet at level that we are comfortable including these as scoring functions. Those physics-based methods that are more rigorous, for example FEP+ [75], require an element of specialist control that, again, is not yet suitable for inclusion in the BRADSHAW framework, but may be included in a design workflow as an additional step [76].

As we hope to have demonstrated, BRADSHAW is at a respectable level of competency in the combined processes that comprise molecule design. The aforementioned rapid pace of innovation and improvement in the field of molecule generation, model building and optimisation algorithms means that systems like BRADSHAW will improve from this level. The potential level of capability that can be reached is difficult to predict. However, even the current level of performance raises questions about how the system should best be integrated into the work practice of a lead optimisation team e.g. is it as a complement to the creativity of the team, or should the automated system become the fundamental workhorse for the team which is complemented by suggestions and decisions by the human supervisors? We feel these answers will become clearer as the platform is further tested in prospective applied scenarios, which will be the subject of a future publication.

## Electronic supplementary material

Below is the link to the electronic supplementary material.

Supplementary file1 (TXT 14 kb)

Supplementary file2 (TXT 0 kb)
